# An Overview on Thermosensitive Oral Gel Based on Poloxamer 407

**DOI:** 10.3390/ma14164522

**Published:** 2021-08-12

**Authors:** Yabing Chen, Jeong-Ho Lee, Mingyue Meng, Naiyu Cui, Chun-Yu Dai, Qi Jia, Eui-Seok Lee, Heng-Bo Jiang

**Affiliations:** 1 Stomatological Materials Laboratory, School of Stomatology, Shandong First Medical University & Shandong Academy of Medical Sciences, Tai’an 271016, China; 17861521896@163.com (Y.C.); mengmingyueaa@126.com (M.M.); Cuinaiyuuu@outlook.com (N.C.); MsDaicy@163.com (C.-Y.D.); aqms-happy@outlook.com (Q.J.); 2 Department of Oral and Maxillofacial Surgery, Graduate School of Clinical Dentistry, Korea University, Seoul 08308, Korea; heaven5454@hanmail.net

**Keywords:** poloxamer, thermosensitive hydrogels, thermoreversible gel, drug release, oral disease drug

## Abstract

In this review, we describe the application of thermosensitive hydrogels composed of poloxamer in medicine, especially for oral cavities. Thermosensitive hydrogels remain fluid at room temperature; at body temperature, they become more viscous gels. In this manner, the gelling system can remain localized for considerable durations and control and prolong drug release. The chemical structure of the poloxamer triblock copolymer leads to an amphiphilic aqueous solution and an active surface. Moreover, the poloxamer can gel by forming micelles in an aqueous solution, depending on its critical micelle concentration and critical micelle temperature. Owing to its controlled-release effect, a thermosensitive gel based on poloxamer 407 (P407) is used to deliver drugs with different characteristics. As demonstrated in studies on poloxamer formulations, an increase in gelling viscosity decreases the drug release rate and gel dissolution time to the extent that it prolongs the drug’s duration of action in disease treatment. This property is used for drug delivery and different therapeutic applications. Its unique route of administration, for many oral diseases, is advantageous over traditional routes of administration, such as direct application and systemic treatment. In conclusion, thermosensitive gels based on poloxamers are suitable and have great potential for oral disease treatment.

## 1. Introduction

**Figure 1:**
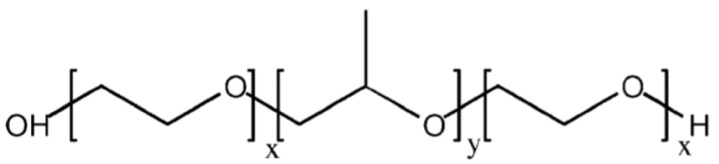
Poloxamer formula: X and y are the lengths of polyepoxyethane (PEO) and polyepoxypropane (PPO) chains. Adapted with permission [2].

Poloxamers are synthetic nonionic triblock copolymers (PEO–PPO–PEO) of polyethylene oxide (Figure 1) [1]. Among these, poloxamer 407 and poloxamer 188 (P188) have been widely used in research of drug delivery systems in recent years.

The weight ratio of oxirane to 1,2-epoxypropane in poloxamer 407 is 7:3, with an average relative molecular mass of 12,600 (9840 to 14,600). The average molecular weight of P188 is 8400 (7680 to 9510) [3,4,5]. As the aqueous solutions of poloxamer 407 and P188 at a certain concentration have reverse temperature-sensitive properties, the concentrated aqueous solution of these poloxamers forms a reverse temperature-sensitive gel. These hydrogels maintain a fluid state at low temperatures, but at body temperature (or room temperature), they become a semisolid gel, and when the temperature decreases, they return to their original fluid state. In this manner, the gelling system is maintained at a local level for a considerable duration, and the drug release is controlled and prolonged. Owing to its good water solubility, low biotoxicity, low stimulation for organisms, and good biocompatibility, it is widely used in the field of biomedicine, especially for oral cavities. For example, using poloxamers, an in situ gel preparation containing moxifloxacin and simvastatin was prepared as a transport drug system to treat periodontal disease. It is suitable for self-administration of an in situ gel for the treatment of oral mucositis [2,6,7,8,9,10,11,12].

As a result, there has been increasing interest in the use of temperature-sensitive hydrogels for various applications. In the past two decades, the number of biomedical papers related to poloxamer hydrogels has exponentially increased [4].

As an ideal drug delivery carrier, thermosensitive gels are widely used in the biomedical field. For example, it has shown great potential in the study of prolonged drug release for ocular administration and the treatment of allergic rhinitis [13,14]. Another formulation which incorporates glass containing Ho_2_O_3_ exhibits suitable properties as theragenerative material for bone cancer treatment [15].

We herein summarize the characteristic properties of poloxamer and its function as a thermosensitive gel as well as cover its role in the delivery of biological therapeutic molecules. Thermosensitive gels have been studied as a scaffold for encapsulating dental pulp stem cells for cell delivery in tissue engineering, buccal applications for oral cancer therapy, intratumoral injection for anti-tumor therapy, etc. [16,17,18]. There have been several studies on its application in the oral cavity but no comprehensive summary. In a previously published article an overview of poloxamer 407 based hydrogels was given and it describes the various applications of hydrogels in different formulations of mucosal tissues like rectal, vaginal and buccal etc. [1]. In this review, we describe in-depth applications of poloxamer 407 in the oral cavity. Therefore, this review is focused on the role and application of poloxamers as a drug transport system in various oral disease treatments.

## 2. Subjects

### 2.1. Poloxamers

#### 2.1.1. Characteristics

The properties of poloxamers depend on the length of the chain on each block; the chemical structure of the polymer gives rise to the polymer amphiphilic and surface active characteristics. With the increase in temperature above the critical cementitious temperature [19], the sol-gel transition of their aqueous solution will occur. In addition, hydrophilic and hydrophobic monomers coexist in the block copolymers and form ordered structures in the solution; the most common structures are micelles. The formation of micelles indicates a unique characteristic, namely, thermal gel properties [20]. Among these characteristics, micellization is the first step of gelling, and the process of micellization is dependent on two key parameters: critical micelle concentration (CMC) and critical micelle temperature required to obtain micelles (CMT). When CMT is higher than the CMC, aggregation occurs. The CMC value is not constant and can shift with the varying environment, CMC value in a given medium is dependent on temperature, pressure, and other factors. Micelles only form above CMT. The micelle process is mainly caused by the hydrophobic-block PPO, which is proven by the inverse ratio between the CMC value and PPO unit number [21]. Owing to the difference in properties between PPO and PEO, the CMC of poloxamers will decrease with an increase in temperature to minimize the effect of poloxamers on drugs [22]. Table 1 describes the properties of most common poloxamer poly(ethylene oxide)-b-poly(propylene oxide)-b-poly(ethylene oxide) (PEO–PPO–PEO) copolymers. Poloxamers are also known as Pluronics, and a special nomenclature was introduced which consists of a letter that indicates the morphism of each copolymer: L (for liquid), P (for paste), or F (for flakes). This is followed by the molecular weight of PPO (first one or two digits) and PEO weight fraction (last digit) [4].

#### 2.1.2. Micellization of Poloxamers

**Figure 2:**
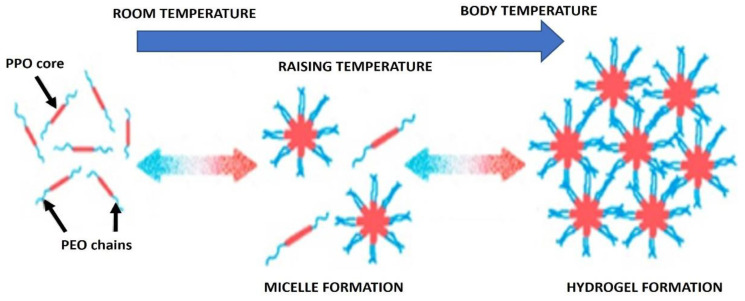
Schematic of the hydrogel formation. Adapted with permission [4].

The properties of poloxamer aqueous solutions have been studied in depth and comprehensively reviewed owing to the unique behavior and benefits of poloxamers. In aqueous solutions, the amphiphilicity of copolymers leads to the self-aggregation of macromolecules into micelles [23]. The core and shell of the micelles are composed of a hydrophobic block and a hydrophilic unit, respectively (Figure 2). They are nanostructures, with a size of usually 10–200 nm, occurring under CMC and CMT. The CMC value of the poloxamer solution decreased with an increase in temperature and PEO chain number, indicating that the polymer with a larger hydrophobic domain PPO would form micelles at lower concentrations and temperatures [24].

#### 2.1.3. Rheological Properties of Poloxamers

Rheology is the study of the relation between the mechanical phenomena generated by a material under external force and the viscosity of the polymer when it flows. The role and application of poloxamers as a drug transport system in the therapy of oral diseases is outlined. In the study of poloxamer in situ gels, injection capacity and gelation time are among the key factors. Gelation time is defined as the time required to change from liquid to gel during injection at a fixed temperature (36.5 °C). The prepared preparation is transferred to a syringe, usually using a 20-gauge needle to fix the volume and inject the in situ gel into the affected area. Below the injectability or gelation threshold, the solution can easily pass through the syringe, whereas above the injectability threshold or gelation threshold, it is difficult for the solution to pass through the syringe and administration is difficult [10,11,25]. Therefore, studying the rheological properties of poloxamers can provide a relevant basis for studying the preparation of its gel formulations.

A temperature-sensitive polymer system comprising poloxamer 407 (PX) and poly(acrylic acid) (PAA), which showed relatively stable viscosity values at low shear rates and decreased viscosity at higher shear rates due to partial disruption of the polymer interactions, was studied. PAA has been observed to increase the gelation temperature of PX solutions such that temperature-sensitive polymer systems with shear-dilution properties can rapidly form gels at the body temperature in clinical applications [6]. Akash et al. studied the rheological properties of P407 under storage conditions and exhibited maximum stability in P407 gel at 4 °C. The in vitro leukocyte receptor antagonist (IL-1Ra) was tested in vitro. The release mode is constant and does not affect the rheological properties of P407 under storage conditions (4 °C). These studies suggest that the P407 gel can adequately support the possible shelf life and stability of IL-1Ra under specific protein and drug storage conditions. In addition to specific rheological properties, the hydrogels prepared by P407 are susceptible to the presence of temperature, concentration, and other compounds [26]. Cristiano et al. studied the flow profiles of hydrogels with different nanosystems prepared using different concentrations of P407 as a function of the applied shear rate, and their viscosity decreased as the shear rate increased. When a specific shear rate value was reached, the viscosity decreased abruptly, followed by a linear decrease in initial and final viscosity. The viscosity value increased with increasing poloxamer concentration. The rheological profile was not affected by the P407 concentration [27]. P407 is a Newtonian fluid at low temperature and a non-Newtonian fluid at high temperature [3]. Fakhari et al. evaluated the rheological and analytical properties of purified P407. Compared with the unpurified P407, purified P407 recorded a higher viscosity value, and the purified P407 undergoes a sol-gel transformation at lower temperatures [28]. Although there have been a few systematic studies on rheology in China, the study of a poloxamer’s rheological properties has an important role in the fundamental research of poloxamers.

#### 2.1.4. Poloxamer 407

Poloxamer 407 (P407) is a polydisperse mixture of triblock copolymerization with a molecular weight of 12.6 kDa. Each molecule has a central hydrophobic PPO block and two hydrophilic PEO on both sides, of which polyethylene accounts for 70%. The copolymer with polyoxyethylene accounting for more than 30% is easy to dissolve in water regardless of its molecular weight. Thus, P407 can be easily dissolved in water. In addition, the aqueous solution containing P407 has good thermal reversibility. The molecular structure of P407 is surrounded by the hydrated layer at low temperatures, and the increase in temperature will cause a hydrogen bond fracture between the solvent and the hydrophilic chain of the copolymer. This process is conducive to the hydrophobic interaction between polymers and the formation of micelles that promotes the gelation process.

**Figure 3:**
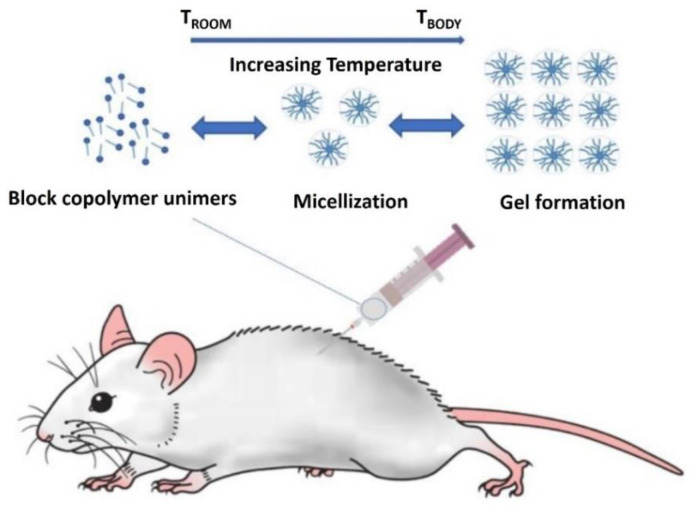
Schematic of in situ gelation mechanism of thermoresponsive P407 aqueous solution. The temperature rise first causes the micelles of P407 to be rearranged into a cubic structure and subsequently form a hexagonal structure. Adapted with permission [1].

The gelation mechanism of the thermoresponsive P407 aqueous solution is that the increase in temperature first causes the micelles of P407 to be rearranged into a cubic structure and then form a hexagonal structure (Figure 3). By studying the P407 formulation, we know that the formulation with 15–30% concentration can appear as a gel at body temperature. The thermosensitive gel based on poloxamer 407 has the characteristics of temperature sensitivity, i.e., at room temperature, it is liquid, whereas it has a gel form at body temperature. This advantage makes it the most attractive drug carrier. When a temperature-sensitive gel based on P407 is applied to the drug delivery site, the drug concentration needed for the lesion will be maintained for a considerable time, thereby reducing the necessary dosage and frequency of application, reducing the side effects of the drug, and increasing patient compliance [29]. In addition, owing to the special chemical structure of P407, the gel has two affinity properties, making it a useful surfactant; it has therefore been applied in many industrial applications. P407 also has good solubilization ability and low biological toxicity [30]. In addition to good drug release ability, it has good compatibility with a variety of biological molecules and chemical reagents [29]. Therefore, it can be used as a solubilizer, emulsifier, or stabilizer and can be administered through local routes. Owing to its solubility, biocompatibility, and non-irritation to biofilms, P407-based thermosensitive gel can be applied to mucosal drug delivery systems. According to the data, P407 is conducive to the dissolution of hydrophobic molecules and promotes their complete and rapid dissolution in the medium [31]. Moreover, thermosensitive gel can be gelatinized at physiological temperatures and adhere to the mucosa of the lesion, thereby improving the retention time of the original flavor.

In addition, owing to the different concentrations of poloxamer, their properties are different hence, they have the characteristics of stimulating sensitivity. They can change their structures according to pH, temperature, among other parameters; this property allows for the preparation of different thermosensitive gels. These differences also change the characteristics of the body and interact with cells and cell membranes as well as design innovative nanodrugs and new organisms. These materials offer great potential. Recently, these gels have been used as substrates for repair and regeneration of various tissues and organs as well as successfully applied to scaffold-forming materials. The latest treatment research shows that gelatin has been used as a 3D printing material owing to its gelation characteristics such as high porosity, rapid gelation, shape maintenance, and immunity [32].

Although P407-based thermosensitive gels are biocompatible, they still tend to be eliminated rapidly in a physiological medium owing to their mechanical strength. Therefore, further research is necessary to perfect the thermosensitive gel. In addition, research data shows that the rate of gel disintegration decreases with an increase in gel concentration. Therefore, when the gel concentration is higher, the gel is more difficult to remove at the lesion site, which is unfavorable to the lesion [33]. The residual gel may block the re-attachment of periodontal tissue. According to the existing research data, low concentrations of P407 do not show cytotoxicity, but there are no specific data to indicate whether high concentrations of P407 show cytotoxicity [34].

### 2.2. Research on Thermosensitive Gel

#### 2.2.1. Preparation of Thermosensitive Gel

The preparation of thermosensitive gel is based on the solubilization of water at the starting point and is dissolved in deionized water at 4 °C until it forms a clarified solution [13,35,36]. In this process, continuous slow stirring is needed to avoid foam formation [24]. Most experimental studies show that the magnetic stirring method has advantages [37]. The method for preparing a thermosensitive gel is to increase gel solvation and remove hydrogen bonding and sample changes. It can better handle the sample, easy to prepare 15–30% gel solution, and the gel characteristically prepared by the auxiliary action of additional additives are liquid at approximately 4 °C, showing a semisolid state at the body temperature [12,38,39]. The drug can keep the drug in gel for a considerable duration and maintain a good effective concentration in the pathogenic part.

#### 2.2.2. Formulation of Temperature-Sensitive Gel Based on Poloxamer 407

In the development of local application of drugs, we should first consider the local physiological conditions, in addition to the retention and release capacity of drugs. Therefore, P407 is not only needed in drugs but also in various additional additives to ensure adaptation to our product requirements. The micellization of the copolymers was adjusted by adding additives. By adding strong crosslinking bonds with poloxamers through additives, some will increase the sol-gel transition temperature, such as hydrochloric acid [40]. Others will reduce the sol-gel transition temperature, such as sodium chloride [41]. To adjust the sol-gel transition temperature, P407 and other poloxamers were used, especially P188 [42]. The gel temperature requirement for local oral administration is 37 gelatin, and it should exist as a liquid at room temperature. However, only gel prepared using poloxamer 407 cannot meet this requirement. The PEO of P188 is approximately 80% and that of PPO is approximately 20%. The study shows that the larger the proportion of nonionic surfactant polyoxyethylene segments, the higher is the hydrophile lipophilic balance value. Therefore, P188 has the properties of being fat soluble and water-soluble. It can be dissolved in the aqueous phase and distributed evenly in the carrier material in the form of molecules, thereby forming a uniform pore structure on the surface of the carrier material; moreover, the addition of P188 can also improve the drug release from the gel for the sake of improving the effective drug concentration in oral lesions. According to the data, the mixture of P407 (15% *w*/*w*)/P188 (15% and 20% *w*/*w*) is the best for obtaining the proper gelling temperature [1].

##### Formulation of Poloxamer 407 Temperature-Sensitive Gel

In addition to P407, several other materials can be used to prepare gels such as natural polysaccharides, hyaluronic acid (HA), chitosan, alginate, and carbomers. These materials form gels that each have the advantage of being mixed with the gels of P407 to form a composite hydrogel. The composite hydrogel shows the properties of its individual components, depending on the physicochemical properties of its components and the structure and interaction of the materials. The resulting hydrogel can be combined to avoid disadvantages, achieve different disease treatment purposes, and solve different problems.

##### Carbomers

Carbomers are macromolecular polymers of acrylic acid-bonded allyl sucrose or pentaerythritol allyl ether. The carboxylic acid group (–COOH) should be 56.0–68.0% as a dry product [43]. Acrylic acid cross-linked resins obtained by crosslinking with an acrylic acid such as pentaerythritol are a class of very important rheology regulators, a well-known pH-dependent polymer that still exists as a solution at acidic pH but forms low-viscosity gels at an alkaline pH. They are mainly used in liquid or semisolid pharmaceutical preparations such as gels, suspensions, and emulsions as thickeners, thereby altering the flow of substances, wherein carbomers are used to modulate the gel strength and bioadhesion. Sometimes, the gelling temperature is slightly elevated due to the action of the drug that can be reduced by the use of carbomers [44]. When applied to moxifloxacin hydrochloride in situ gels for periodontitis treatment, the in situ gels provide drug release at a controlled rate through direct access to the target site, thereby reducing side effects and thus improving patient compliance [11]. Moreover, as they are used in the nose to treat colds, carbomer gel inhibits the replication of human rhinovirus in the epithelium by creating a physical barrier to the human nasal epithelium and reducing viral cation binding sites while preventing the virus from crossing the cell surface to the nasal epithelium [45].

Carbomers as bioadhesive polymers can enhance the gel strength of temperature-sensitive hydrogels. Chen et al. selected Carbomer 940 as a bioadhesive polymer to enhance the gel strength of P407/P188 thermosensitive hydrogels. P407, P188, and carbomer 940 were used to prepare thermosensitive composite hydrogels (TCH). These results suggest that TCH should be used in transdermal delivery systems and that TCH is a promising new carrier for transdermal drug delivery [46]. In addition, Garala et al. successfully formulated chlorhexidine hydrochloride, a broad-spectrum antimicrobial agent used in the treatment of periodontal disease, into a temperature-sensitive in situ gel using P407 and carbomer 934 as gels [47]. Its transparency, pH, and drug content were satisfactory and all preparations showed sustained drug release for up to 6 h. The gel formed by mixing a carbomer with a poloxamer is more advantageous and the drug release is more stable, favoring disease treatment.

##### Hyaluronic Acid

HA is a high-molecular-weight glycosaminoglycan (GAG) comprising a disaccharide non-sulfated unit repeat of D-glucuronic acid and N-acetylglucosamine. HA is a very important biomolecule because it is one of the main components of the extracellular matrix and is widely distributed in various tissues, including skin [48], synovial fluid, cartilage, tendons, eyes, and most body fluids [49]. As HA is involved in several biological processes related to morphogenesis and tissue healing as well as its biocompatibility, biodegradability, and non-immunogenicity [50], hyaluronic acid acts by promoting the enhancement of gel structure through hydrogen bonding between polymers. Experiments have shown that the interaction between the two compounds promotes a large number of interactions between the obtained gel and the mucin of the oral mucosa, making the release of the drug a controlled factor. Thus, HA has been extensively studied as a powerful tissue engineering biomaterial over the past decades [51].

HA is an essential glycosaminoglycan for the extracellular matrix of periodontal tissue, and most cells can produce it at multiple stages of their cell cycle [52]. It is involved in tissue repair and wound healing by stimulating cell proliferation and by interacting with several growth factors. Current studies have used HA gels for papillary reconstruction, but adequate evidence and studies are still lacking; more experimental evidence is needed to evaluate the use of injectable HA gels for the treatment of interdental papillary loss. In addition, hyaluronic dermal fillers are the most popular non-permanent injectable materials that physicians can use today to correct facial soft tissue defects [53]. This material offers an effective, non-invasive, non-surgical alternative to correct facial contour defects owing to its tremendous ability to bind water and its ease of implantation. The formation gel can be used to plump up the lips or reshape the atrophied jaw and contour it for a youthful appearance by lifting and tightening the aging sagging skin tissue. HA promotes the enhancement of the gel structure through hydrogen bonds between polymers. The experiments show that the interaction between the two compounds promotes the interaction between the gel obtained and mucous membrane of the oral mucous membrane, which makes drug release a controllable factor. HA also plays a role in the treatment of alveolar bone restoration by promoting cell migration and inducing the proliferation of undifferentiated mesenchymal cells to osteoblasts [54].

The addition of HA to poloxamers to form a temperature-sensitive gel can optimize the gel performance, as in the case of Hsieh et al., who developed a gel based on P407 binding with HA to optimize the loading and release of urokinase [55]. The results showed that the addition of HA to the poloxamer gels could change the microstructure, making them more compact and with smaller pore sizes. In vitro toxicity tests showed low toxicity [56], indicating that P407/HA hydrogels are effective and safe materials for future loading of hydrophilic drugs owing to their high safety profile, which does not cause cytotoxic reactions after local injection. Sodium hyaluronate, as an additive to P407 gels, reduces CMT and accelerates the micellization process without affecting micellization strength. When mixed with aqueous P407, the HA-added gels were found to perform better in slowing gel erosion and improving gel properties. The new material was also effective in promoting skin wound healing [57] by enhancing protein accumulation in the wound area, increasing permeability, and preventing bacterial invasion. In addition, its effective moisturizing and breathability ability helps to prevent wound infection and protects the wound, thus avoiding the need for antibiotics [58].

##### Chitosan

Chitosan is a natural copolymer having good biocompatibility, antibacterial activity, and biodegradability [59]. Chitosan improves the mucosal adhesion properties and gel strength of poloxamer gels because chitosan has positively charged amine residues that facilitate interaction with the gel solution to provide the drug with proper mechanical and mucosal adhesion properties [60].

Chitosan temperature-sensitive hydrogels are safe and have good drug bioavailability [61]. It is a natural polymer with good biocompatibility, biodegradability, mucosal adhesion properties, antibacterial and antifungal properties as well as the ability to promote enhanced penetration and corneal wound healing. Gel formation is the result of non-covalent interactions such as electrostatic, hydrophobic, or hydrogen bonding. The polymers form gels without additives alone [16].

The results showed that the cytotoxicity of chitosan temperature-sensitive gels was grade 0, and chitosan-based thermosensitive hydrogels have a great advantage in ophthalmic drug delivery [62]. Chitosan temperature-sensitive gel microspheres containing the drug blocked the sudden release of the drug in the initial phase and later facilitated the slow release of the drug. Cao et al. formulated an in situ gel based on chitosan and poloxamers for the intraocular release of the anti-glaucoma drug timolol maleate [14]. It has bioadhesive properties, and the in vitro corneal permeability studies on goat corneas showed increased permeability of the in situ coagulation system after 4 h compared to eye drops. This is due to the ability of chitosan to increase the permeability of the mucous membrane.

In addition to its effective use for the eye, chitosan can also address oral problems. For example, Madrazo-Jiménez et al. studied a topically applied chitosan gel and evaluated its application in mandibular third molar surgery; it was shown to significantly benefit the healing of the surgical wound [63].

#### 2.2.3. Measurement of Sol-Gel Transition Temperature and Gelation Time

**Figure 4:**
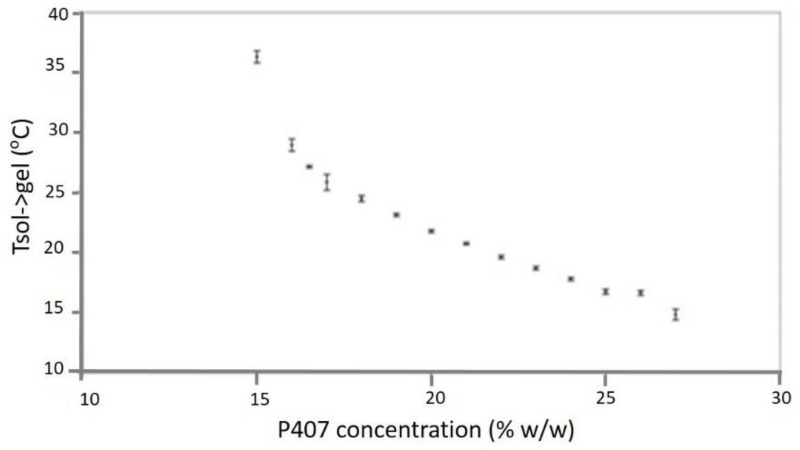
The influence of the concentration of p407 on the temperature of gelation (T_sol_→T_Gel_) (with standard error bar). Adapted with permission [66].

Thermosensitive gels have important characteristics. They undergo a sol-gel transition under certain temperature conditions. The gelation temperature indicates the temperature of the phenomenon [64]. Therefore, the basic prerequisite for developing highly effective viscose preparation is the determination of the sol-gel transition temperature. The concentration of additives, such as P407, also affects the gelation and transformation of thermosensitive gel (Figure 4). The gelation temperature is determined by a magnetic stirrer [40]; that is, a poloxamer solution is added to the transparent vial of a constant-temperature water bath at a low temperature; then, the magnetic rod and the digital heat sensor are immersed in the solution [65]. The solution is then continuously stirred and heated. When the magnetic rod stops, gelation is indicated [41], and the displayed temperature represents the gelation temperature. Another method is the use of a rotary rheometer for measurement. The gelling temperature is evaluated by an oscillating measurement at a constant frequency value (most commonly used frequencies are 0.01 and 1 Hz) over a temperature range that includes the physiological temperature.

Gelation time refers to the time of rapid gelation of drug preparations at body temperature after administration; this is an important factor. The determination of gelation time is important for gel removal from the injection site and for evaluating drug retention time. Gelation time is usually measured by simulating the oral environment and adding gel to the gel.

### 2.3. Drug Release of Poloxamer Temperature-Sensitive Gel

Although the poloxamer-based in situ gelled drug delivery system is attractive, its characteristics of rapid erosion and rapid drug diffusion make it difficult to achieve sustained drug release. The composition, concentration, and properties of poloxamer are the most important factors determining the release rate of in situ gelling drugs [67]. The hydrophobic gel matrix has a compact micellar structure; saliva washing and other factors cause slow drug diffusion and gel erosion. The drug release of the poloxamer-based in situ gel will be controlled by both the drug diffusion rate and the gel erosion rate [68]. The release rate varies according to the composition ratio of the gel matrix and the physical and chemical properties of the drug contained. Therefore, as poloxamer-based in situ gel drug delivery systems are susceptible to erosion and the residence time at the drug delivery site is too short, their application as sustained/extended release platforms is also limited. Therefore, we are actively studying different component ratios to solve these problems and improve the poloxamer-based in situ gelation drug delivery system [67].

#### 2.3.1. Diffusion of Drugs Using Thermosensitive Gels Based on Poloxamers

As the gel particles form a continuous pipe-like water path in the microstructure, drug diffusion plays a primary role in the poloxamer-based gel. There are certain differences in the physical and chemical properties of the drugs carried in the gel, such as the difference in molecular weight, lipophilicity, and hydrophilicity, which will lead to different drug diffusion characteristics [67]. On the one hand, due to the increased lipophilicity of the drug, the alkyl chain becomes longer, leading to an increase in affinity for the micellar nucleus, which is inherently lipophilic, thereby slowing down the diffusion of the gel. On the other hand, the affinity of the small molecular weight aqueous drugs often exist in water pathways, which quickly spread to the surrounding environment [67,69]. Similarly, the supramolecular arrangement of the gel matrix, volume of the micelles, entanglement of the micelles, and size of the water passage gaps will also affect the drug diffusion rate of the in situ gel system [70,71].

#### 2.3.2. Hydrate Decomposition of Temperature-Sensitive Gel Based on Poloxamers

In the presence of excess aqueous medium, the poloxamer micellar filling structure rapidly decomposes, and the gel structure will be destroyed at this time. The micelles on the surface dissolve and release the medium, resulting in gel hydrate decomposition and drug release [38,72]. The following two situations are mainly based on the release mechanism of gel hydrate decomposition. The first is when the molecular weight of the drug-carrying molecule is large and/or lipophilic. The second is when the mechanical strength of the formed gel is insufficient to resist hydrate decomposition; the drug will be released as the gel disintegrates [73,74].

#### 2.3.3. Additives Promote Drug Release

The methods used to improve the in situ gel system of poloxamer have mainly involved mixing poloxamer with various other polymers in different ratios. The interaction between the poloxamer and the added polymer changes the structure of the gel molecules, thereby significantly affecting the porosity, mechanics, and rheology of the gel. Hydrophobic bonds, hydrogen bonds, and supramolecular interactions (intermolecular forces bound to polymers) together constitute an interaction that changes the structure of the gel. The drug release properties of HA [7], chitosan [75], alginate, cellulose derivatives [76], sodium chloride, and other substances widely used in the poloxamer in situ gel system change the gel release characteristics. Due to the hydrogen bonding between HA and poloxamer, mixing HA into poloxamer solution can increase the gel strength and reduce the network porosity, thereby maintaining the release of the drug and reducing the initial explosiveness of the drug release [7]. In addition, adhesion and residence time increased. Chitosan and poloxamer form a pH-reactive hydrogel; a sol-gel transition caused by a change in pH. The blending of chitosan and poloxamer gel increases the gel strength by forming an inter-micellar bridge, thereby reducing the drug release rate. In addition, chitosan enhances the adhesion of the gel and prolongs the residence time in the mouth [77,78]. The type and concentration ratio of chitosan determine the intensity and direction of the change. Cellulose derivatives and poloxamer can also increase the strength of the gel and reduce the erosion rate of the gel in the oral cavity to slow the drug release [76]. Certain cellulose derivatives such as hydroxypropyl methyl cellulose (HPMC) and hydroxyethyl cellulose (HEC) enhance adhesion and thus increase the residence time of the drug gel in the oral cavity [79]. These changes are also controlled by the type of cellulose derivative and specific gravity of the gel. Alginate is an anionic polymer with strong adhesion biocompatibility. The mixture of alginate and poloxamer can reduce the erosion rate of the gel in the oral cavity, achieve the effect of increasing the residence time of the gel, and slow down the drug release. When sodium salt is involved in the formation of poloxamer gel, it can improve the mechanical strength and adhesion of the gel [80]. However, studies have shown that due to the high hydrophilicity of sodium salts, when exposed to a water environment after a certain period of time, they often exhibit a “pore-forming effect”. Owing to this high hydrophilicity, sodium salt easily diffuses into the oral cavity from the gel water channel. The precipitated sodium salt will leave pores in the gel, and external liquids such as saliva in the oral cavity can more easily enter the gel matrix, thereby increasing the diffusion and erosion rate of the gel.

### 2.4. Application of Thermosensitive Gels in the Oral Cavity

**Figure 5:**
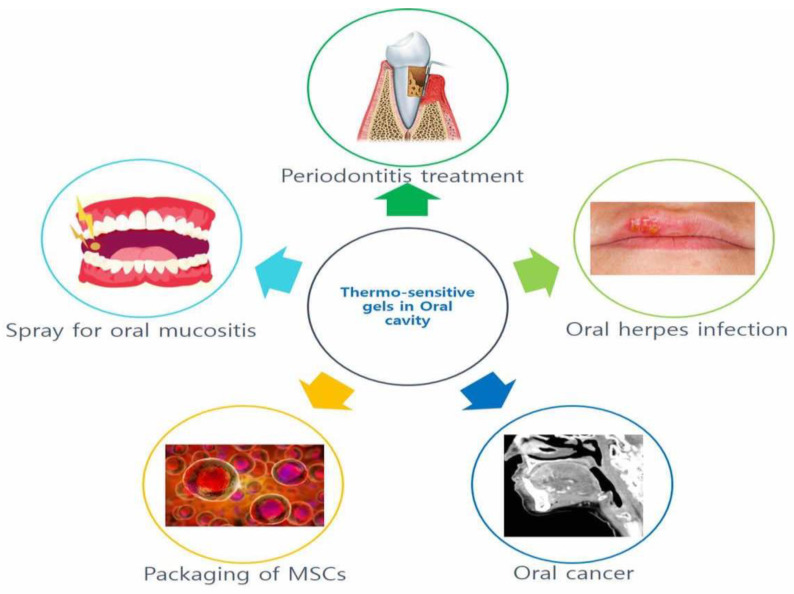
Thermosensitive gels for the oral cavity.

The special physiological structure and environment of the oral cavity lead to difficulties in oral administration; therefore, a drug carrier is urgently needed to solve the problem of oral administration. P407 and carbomer can form a temperature-sensitive biogel binary mixture having good mechanical and mucous membrane adhesion characteristics; this biogel mixture can be designed as a drug supply platform for an oral implantable drug delivery system (Figure 5). Topical administration is usually used to treat local diseases such as periodontitis, oral rash, and other oral diseases. The main advantage of this drug delivery is that it can directly deliver the bioactive agent to the affected area, maintain the required drug concentration for a considerable period, and ensure good retention at the application site. Below the sol-gel temperature, the fluidity and compression characteristics of the formulation are extremely low. In contrast, for conditions above the sol-gel transition temperature, these formulations exhibit a wide range of viscoelasticity, mechanical, and mucoadhesive properties, which will facilitate their application in the desired parts of the oral cavity. The high elasticity and mucous membrane adhesion will make the formulation containing P407 useful as a platform to control local drug delivery in the oral cavity. At present, temperature-sensitive gels have been widely used to treat various periodontal diseases, and there are many types of preparations. For example, the study of moxifloxacin hydrochloride in situ gel treatment of periodontitis, injectable in situ curcumin gel for the treatment of periodontal pocket, temperature-sensitive gel for the treatment of oral herpes infection, and temperature-sensitive gel as a clinical case of the stent packaging system.

#### 2.4.1. Thermosensitive Gel for Periodontitis

Based on the preparation of poloxamer thermosensitive oral gel, the main consideration is determined as the addition of drugs into the thermosensitive oral gel containing poloxamer, to better treat periodontitis. Periodontitis is caused by a microbial biofilm that leads to host reaction and periodontal tissue destruction.

Controlling inflammation is considered a potential mechanism for periodontitis treatment. There is strong evidence that endogenous anti-inflammatory and solubilizing processes inhibit chronic inflammation and accelerate wound healing by stimulating tissue regeneration [81]. Therefore, we hypothesized that when a drug with a suitable carrier can be stored in the periodontal pocket for a long period with continuous drug release, the drug carrier system is better than direct release. Thermosensitive gels have unique advantages across all drug carriers [34]. Because they are in a liquid state at low temperatures, they can easily be injected into the periodontal pocket and form a solid gel at body temperature to facilitate the continued release of the drug [4,10,23,82]. The average pocket temperature is 36.6 °C ± 0.4 °C for a temperature value higher than 30 °C, a sharp increase in the P407 solution viscosity can be observed, indicating that P407 expresses different degrees of gelling ability with a temperature change. The use of thermosensitive gel, which is characterized by the gel temperature near the pocket temperature [6,10], represents the effectiveness of the drug delivery system for oral administration. In particular, the thermosensitive gel exhibits a semisolid state at room temperature. Its low viscosity ensures that the drug is easy to use, can be evenly distributed in the periodontal pocket, and adequately infiltrates the drug solution into the periodontal pocket. In addition to ensuring the therapeutic effect of drugs in the oral cavity, maintaining the required concentration of drugs in the administration site for a long period is necessary [37,83].

**Figure 6:**
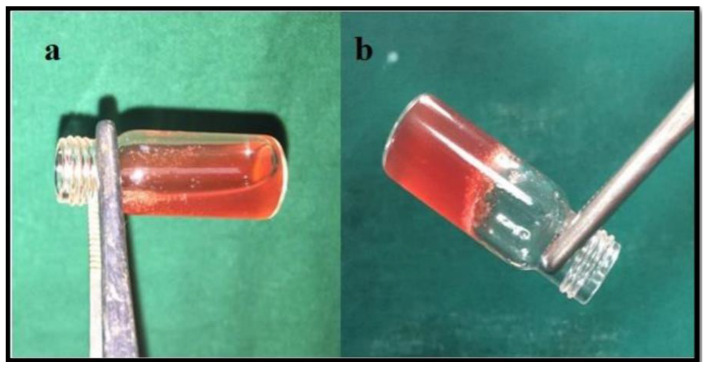
Representative photograph of CJC thermoreversible gel at (**a**) room temperature (24 °C) and (**b**) gelation temperature (32 °C). Adapted with permission. (Copyright 2021) [86].

According to research, a temperature-sensitive gel based on P407 exerts an immense influence on the treatment of periodontal disease. Swain et al. formulated moxifloxacin hydrochloride thermosensitive smart gel using a combination of P407 and gellan gum for subgingival delivery to extend its residence time in the infected cavity to improve the local effect of periodontitis (Table 2). There is no indication of incompatibility between the drug and gel, and it retains its antibacterial activity [11]. In addition, Sheshala et al. used polylactic acid (based on poloxamer) and chitosan as heat-sensitive and adhesion polymers to prepare in situ gel formulations including moxifloxacin for periodontitis therapy. Previous experiments have shown that all formulas have appropriate viscosity and can easily pass through the syringe, thereby also prolonging the retention time in the affected area. All formulations are transparent, and the pH value does not irritate the oral mucosa [10]. Thus, the researched poloxamer-based gel formulation is suitable for periodontal application. Furthermore, as a polyphenolic compound, curcumin can exert an anti-inflammatory effect by reducing the inflammatory mediator of periodontitis. Local administration of oral curcumin as a target can avoid extensive intestinal and primary X. Over-metabolism prolongs the duration of action of the drug, thereby maximizing its local effect. Therefore, the local introduction of curcumin into the system to maintain its chemical stability and improve its solubility is a promising method for periodontitis treatment. Nasra et al. prepared in situ curcumin gel with 30% P407 and 1% carbopol P934 to explore the injectable in situ curcumin gel for periodontal pocket treatment. Clinical research on periodontitis shows that in this drug delivery system, the probe depth and bleeding index of curcumin can be reduced and therefore, can be used as a new method for the treatment of periodontal disease with better patient compliance [84]. Bansal et al. prepared in situ gels containing metronidazole and levofloxacin for in vitro studies and antimicrobial experiments to investigate their feasibility for periodontitis treatment. The experiments showed no interaction between the drug and the carrier gel. The formulation containing 1.5% w/v Cs was injectable and close to the oral pH without any potential irritation. The duration of drug release could be extended up to 48 h, and the high viscosity made it easier to store the gel in the periodontal pocket for a long period of time. In addition, compared with carrier gels, in situ gels exhibit stronger antibacterial effects. Therefore, the authors speculated that the prepared in situ gel could be used to treat deep infections in periodontal pockets [37]. In situ injectable gels prepared with P407 and aerosil that can be used in periodontal pockets, doubly control metronidazole benzoate and serratiopeptidase delivery. The developed gel has good injectability and has better access to pathogenic bacteria deep in the periodontal pockets; this is more comfortable for patient use. The in situ gel prepared with P407 as a drug delivery system can maintain an effective dose of drug for a longer period of time. As the drug permeates, it causes the poloxamer and aerosil matrix to swell; the swollen gel is better anchored in the periodontal pocket [85]. Although chlorhexidine gluconate (CHX) has a wide range of clinical applications for the treatment of periodontitis, researchers have focused their research on phytotherapy due to its numerous side effects. American cranberry has a special polyphenol composition and therefore, excellent antibacterial potential. Therefore, Rajeshwari et al. prepared a thermally reversible gel containing cranberry juice concentrate (CJC) using P407 to evaluate its antibacterial properties against periodontal pathogens and its biocompatibility. The study was performed by controlling the poloxamer concentration such that the CJC could be released from periodontal pockets in the subgingival temperature range, and the drug activity was retained (Figure 6). The original gel has good adhesive strength, thereby prolonging its retention time in the periodontal pocket, which in turn can prolong the release of the drug; thus, the prepared thermoconin gel has good stability [86].

#### 2.4.2. Other Applications of Thermosensitive Gels in the Oral Cavity

##### Thermosensitive Gel for Oral Herpes Infection

Oral herpes is a viral infection of the mouth and lips primarily caused by a specific type of herpes simplex virus. The virus can cause painful sores on the lips, gums, tongue, inside the cheeks or nose, and on the face. It can also cause swollen lymph nodes, fever, and muscle aches [87].

Herpes simplex virus (HSV) is a DNA virus [87,88]. These herpes viruses enter the body through small cuts, abrasions, and rupture of the skin or mucous membranes. The incubation period for a herpes simplex infection is approximately three to six days. Transmission of the virus occurs from person to person, and HSV virus multiplies in human cells. One of the steps in its proliferation is assuming control of the nucleus of human cells to change their structures. Sores occur because as the virus matures, many HSV particles rupture the cell membranes, resulting in the affliction of herpes [89].

Chaudhary and Verma developed an oral mucosal drug delivery system for topical and systemic delivery of acyclovir for the treatment of oral herpes infections HSV. The use of a temperature-sensitive gelling system increases the duration of drug action, thereby increasing acyclovir bioavailability in the oral mucosa. Drug release studies have shown sustained release properties. All preparations showed sustained release properties. The first-pass effect was avoided by in situ delivery of the drug gel formulation through the oral mucosa. Therefore, thermally sensitive in situ gels provide a reasonable choice for local and systemic administration and ultimately enhance the bioavailability of drugs. This method can be used to locally treat oral herpes infections by improving patient compliance [90].

##### Application of Thermosensitive Gels for Oral Cancer

Oral cancer is the sixth most common malignant tumor in the world with poor prognosis. Oral squamous cell carcinoma (OSCC) is the main type of oral cancer. Its incidence is related to excessive drinking, smoking, exposure to ultraviolet radiation, immunosuppression, and age. These risk factors can lead to the transformation of healthy oral mucosa cells into malignant proliferative lesions before carcinogenesis and then develop into OSCC. Although cancer can occur in different areas of the oral cavity [91,92], the prognosis has not improved with the progress of conventional treatment, and more adverse reactions and complications occur post-treatment. Therefore, a safer and more effective treatment is urgently needed to improve prognosis and reduce adverse reactions and complications post-treatment. Studies have shown that the preparation of curcumin made of the poloxamer-based thermosensitive gel has a great therapeutic effect on OSCC [17]. Curcumin also demonstrates excellent biological activity, which also has great advantages for OSCC. The cubic hexagonal colloid cementing system formed by P407 has an ordered structure, which can make hydrophilic and hydrophobic drugs mutually compatible. It can dissolve and deliver the drug to the target site [12], thereby protecting the drug from explaining, increasing solubility, and providing sustained release and penetration, while maintaining bioactivity. When applied to the oral cavity, the P407 curcumin gel has rheological properties, viscoelasticity, and mucosal adhesion, and can increase its retention in the oral cavity [93,94]. In addition to curcumin showing excellent physical and chemical properties for the treatment of oral cancer, the P407-based thermosensitive gel system also shows encouraging results.

##### Temperature-Sensitive Gel Used as the Packaging System of the Bracket

As a treatment, MSCs have shown considerable potential. A common use of stem cells is by injecting them directly into the target tissue. However, such injections cause stem cells to flow back from the injection site and result in the loss of some transplanted cells [95]. The thermosensitive gel can also be used as a scaffold encapsulation system to maintain the survival rate of MSCs while delivering MSCs to the defect site to solve the problems caused by MSCs-mediated tissue regeneration. P407 is an effective and nontoxic scaffold for the encapsulation of dental pulp stem cells (DPSCs) as well as for the control of human bone marrow MSCs [96] that enhances stem cell viability and proliferation. It has a dense tubular and reticular network morphology that contributes to its high permeability and solubility. A wound-healing model was used to compare the wound-healing abilities of a hydrogel encapsulation system containing MSCs and a single injection of MSCs. In contrast, the wound-healing rate was significantly accelerated and skin appendages recovered better with the hydrogel encapsulation system containing MSCs. Moreover, stem cells delivered in hydrogels survive longer and are more efficient than stem cells delivered by injection. Compared with injection-delivered MSCs, gel-encapsulated MSCs could induce stem cells to secrete angiogenic cytokines expressing multifunctional and self-repairing cytokines (Oct4, Sox2, and Klf4) [97]. The angiogenesis of wounds exposed to the gel was significantly enhanced. The insertion of hydrogels interferes with the ability of plasmid DNA to deliver therapeutic growth factors after transcription. In conclusion, temperature-sensitive gels are promising scaffolds for encapsulating DPSCs and can be considered for cell delivery in tissue engineering, and gels containing dental pulp stem cells are promising and nontoxic scaffolds for DPSC encapsulation. Immobilization of DPSCs in P407 gels thus offers a promising approach for tissue engineering [18].

##### Temperature-Sensitive Gel as a Spray for Oral Mucositis

The temperature-sensitive gel can also be used as a liquid spray for application in the oral cavity. P407 or poloxamer 123 as a heat-sensitive polymer in combination with the adhesive polymer polyvinylpyrrolidone and sodium carboxymethylcellulose or chitosan to prepare an in-situ film-forming gel suitable for spraying on the oral mucosa. They allow for local drug release to protect the area of damage caused by the thin-film gel formed after mucosal contact, making self-application easy. In addition, the adhesive capacity of in situ gelation can increase the residence time after coating and form a physical protective barrier in the affected area. Further, it also relieves pain and discomfort caused by tongue movement, food contact, saliva enzymes, and other stimuli [8].

## 3. Summary

In this review, we have summarized the application of temperature-sensitive hydrogels composed of poloxamers in medicine, especially for the oral cavity. A limitation of this review is that as it is a narrative review, although different studies were analyzed and their similarities and differences were considered for this review, there may have been some points missed in the process. However, we can conclude that poloxamer-based thermal gels have good drug-releasing properties, reversible thermal properties, and rheological properties.

In the preparation of gels, various additives need to be used to adjust the copolymer properties to achieve the required characteristics. For example, HA, carbomer, chitosan, hydrochloric acid, sodium chloride, among others, are used to ensure suitable mechanical and adhesion characteristics as well as better control the action time and release of the drug.

P407 and additives can form a temperature-sensitive bio gel mixture, which has good mechanical and mucoadhesive properties and can be designed as a drug supply platform for oral implantable drug delivery systems. The temperature-sensitive gel of poloxamers is mainly related to periodontitis treatment in the oral cavity. By adjusting the ratio, the drug and the aqueous solution can be converted into a gel in the oral cavity and adhered to the oral cavity to achieve continuous administration. It can also be used as a scaffold packaging system to solve the problems caused by MSC-mediated tissue regeneration. In conclusion, the thermosensitive gel based on poloxamer 407 is suitable for the treatment of oral diseases, and its potential for treating oral diseases is considerable.

## Figures and Tables

**Table 1:** Properties of the most common poloxamer poly(ethylene oxide)-b-poly(propylene oxide)-b-poly(ethylene oxide) (PEO–PPO–PEO) copolymers. Adapted with permission [4].

Poloxamer	Pluronic	PEO%	Average Molecular Weight	Melting Point (℃)	Viscosity (Pa·s)	Surface Tension (dyn cm^−1^)	HLB *
P105	L35	50	1900	7	0.375	49	18–23
P108	F38	80	4700	48	0.260	52	>24
P122	L42	20	1630	−26	0.280	46	7–12
P123	L43	30	1850	−1	0.310	47	7–12
P124	L44	40	2200	16	0.440	45	12–18
P182	L62	20	2500	−4	0.450	43	1–7
P183	L63	30	2650	10	0.490	43	7–12
P184	L64	40	2900	16	0.850	43	12–18
P185	P65	50	3400	27	0.180	46	12–18
P188	F68	80	8400	52	1.000	50	>24
P212	L72	20	2750	−7	0.510	39	1–7
P215	P75	50	4150	27	0.250	43	12–18
P217	F77	70	6600	48	0.480	47	>24
P234	P84	40	4200	34	0.280	42	12–18
P235	P85	50	4600	34	0.310	42	12–18
P237	F87	70	7700	49	0.700	44	>24
P238	F88	80	11,400	54	2.300	48	>24
P288	F98	80	13,000	58	2.700	43	>24
P333	P103	30	4950	30	0.285	34	7–12
P334	P104	40	5900	32	0.390	33	12–18
P335	P105	50	6500	35	0.750	39	12–18
P338	F108	80	14,600	57	2.800	41	>24
P402	L122	20	5000	20	1.750	33	1–7
P403	P123	30	5750	31	0.350	34	7–12
P407	F127	70	12,600	56	3.100	41	18–23

* HLB: hydrophilic–lipophilic balance.

**Table 2:** Relevant literature based on Poloxamer 407 for the treatment of periodontitis.

Publication	Components	Advantages
Ganesh P. Swain, Shivani Patel (2019) [11]	Moxifloxacin Hydrochloride, Methyl cellulose, Carbopol P934, poloxamer 407, Gellan gum, Sodium citrate, Triethanolamine, Deionized water.	The formulations extend their residence time in the infected cavity to improve the local effect of periodontitis. There is no indication of incompatibility between the drug and gel.
Ravi Sheshala (2018) [10]	Moxifloxacin HCl, poloxamer 407, poloxamer 188, HPMC E4M, Chitosan in 1.5% *v*/*v* lactic acid, Benzalkonium chloride, β-glycerophosphate.	All formulations have moderate viscosity and prolong the residence time in the affected area. The pH value has no irritating effect on the oral mucosa.
Maha M.A. Nasra(2017) [84]	Curcumin Cur, Pluronic 127, Carbopol P934, polyethylene glycol 400, potassium di-hydrogen phosphate and sodium lauryl sulfate, tri-ethanol amine, PEG 7-glyceryl-cocoate.	In this drug delivery system, the probe depth and bleeding index of curcumin can be reduced and can therefore be used as a new method for the treatment of periodontal disease. It also leads to better patient compliance.
Monika Bansal(2017) [37]	Levofloxacin (LVF), metronidazole (MZ), chitosan, poloxamer 407.	The duration of drug release could be extended up to 48 h, and the high viscosity makes it easier to store the gel in the periodontal pocket for a considerable period. In addition, compared with carrier gels, in situ gels exhibit stronger antibacterial effects.
Pathak, K. and N. Kumari(2012) [85]	Metronidazole benzoate, Metronidazole, serratiopeptidase, poloxamer 407.	The developed gel has good injectability and forms a gel that is more accessible to pathogenic bacteria deep in the periodontal pockets and can maintain an effective drug dose for considerable period.
Rajeshwari H. R(2017) [86]	Poloxamer 407, chlorhexidine gluconate, Carbopol P934. The original gel has good adhesive strength that can prolong its retention time in the periodontal pocket, which in turn can prolong drug release.	

## Data Availability

The data supporting this study are included in the article and have been licensed for use.
